# Prohibiting Headgear for Safety in Amateur Boxing? Opinion of the Canadian Boxing Community: an Online Poll

**DOI:** 10.1186/s40798-016-0043-2

**Published:** 2016-02-11

**Authors:** Philip Dickinson, Philip Rempel

**Affiliations:** Centre Sportif Chat Bleu, 435 rue Beaubien Ouest, 4e, Montreal, Quebec H2V 1C9 Canada

**Keywords:** Amateur boxing, Concussion, Safety, Head injury, Opinion poll, Headgear

## Abstract

**Background:**

In 2013, the Amateur International Boxing Association (AIBA) introduced a rule banning headgear for male-senior open class boxers during competition. The AIBA has defended the rule change as motivated by safety and supported by internal unpublished studies. As a result, in 2018, the AIBA plans to universally prohibit headgear in competition: for all competitors (male and female), all ages and all levels. Within Canada, this ruling has generated controversy in the boxing community, yet there has been no overall measure of opinion.

**Methods:**

To address this, we instituted a voluntary, anonymous, online open-access poll to allow members of the boxing community to express their stance on headgear use in competition.

**Results:**

In total, 636 responses were received. A total of 71.5 % of Canadian respondents believed headgear should be mandatory at all levels. Only 5.8 % agreed that headgear should be prohibited, as planned for 2018. Estimating results on a representative breakdown of boxing membership in Canada, a similar pattern emerged, whereby 68.2 % concurred with mandatory headgear while only 4.95 % supported its prohibition. Parents of boxers were almost unanimously against banning headgear, stating they would change sports as a result. Similarly, only 1.7 % of women believed headgear should be prohibited.

**Conclusions:**

The consensus of the Canadian boxing community largely opposes the rule changes that the AIBA has implemented. The results highlight risks posed to the long-term viability of the sport, if significant grassroots safety concerns are disregarded.

## Key Points

- The large majority of the Canadian boxing community supports mandatory headgear use in amateur boxing.
- Parents of boxers, women, officials and ringside physicians were almost unanimously against the banning of headgear.
- Active boxers were more amenable to conditional removal of headgear than other respondents, but strongly against its prohibition.

## Background

In 2013, the international governing body for amateur boxing, the Amateur International Boxing Association (AIBA), ruled that elite men would no longer be permitted to wear headgear in competition [1]. In addition, the scoring was changed from the previous computer scoring to a 10-point must system similar to professional boxing. In Canada, *elite* has been interpreted to mean any male boxer between the ages of 19 and 40 with more than 10 fights. In 2018, the AIBA plans to universally prohibit headgear from competition (see rule 20.1) [2], which would include all women and children in any competition at any level.

With increased awareness of serious consequences from head injury and concussion in sports [3, 4] and concern for the long-term effects of repeated blows to the head [5], the implementation of these rule changes is confounding. In response to potential concerns, the AIBA has maintained that it is safer not to wear headgear [6]. Thus, an integral part of the controversy arises from conflicting arguments for the safety provided by headgear.

Through the media, the AIBA has referenced an internal study showing reduced injury following headgear removal [7]. Without peer review, however, these claims are problematic. A second study (peer reviewed) referenced by the AIBA [8] to support headgear removal found a short-term increase in bouts stopped at the referee’s discretion after the introduction of headgear. This observation, however, may have resulted from particular caution following ring deaths in pro-boxing that immediately preceded headgear use [9], since the observed increase was temporary. Instead, along with a reduction in knockouts, this study ultimately found that headgear and computer scoring increased safety in all aspects of the sport [8]. In a recent analysis, the impact of punches was systematically examined both with and without AIBA-approved headgear. A significant reduction in impact was observed with headgear, and it was concluded that the use of headgear can “play an important role in reducing the risk of concussion” [10]. Related studies have produced similar results for the reduction of both linear [11] and rotational impact [12] through the use of boxing headgear. In the Zurich Consensus on Concussion in Sports, the authors recommend sport-specific studies to develop protective headgear that is designed in response to the particularities of each sport [3]. This is supported by a recent study that highlights variable protective performance of different types of headgear [13]. In fact, the lead author and senior author from the Zurich Consensus have levelled criticism directly at the AIBA for the headgear ban and for not participating in international discussions on concussion and athlete safety [14].

As a consequence, the boxing community is left with many unresolved questions and concerns. How these opposing sources of information impact opinion within the boxing community, however, is unknown. As a result, it is highly relevant to investigate the sentiment of the boxing community in regard to the rule changes. The prohibition of headgear has been applied internationally for 2 years and, more recently, at the regional level in Canada. We consider the Canadian boxing community a sample of particular interest, considering that the use of protective headgear was first introduced in Canada in 1971, well before international norms were established in 1984 [14]. To measure opinion within the Canadian amateur boxing community, we created an open-access, online poll that was voluntary and anonymous. In this poll, we permitted respondents to outline their preferences for headgear use, identify their demographic characteristics and provide feedback. This study is timely, considering the desire of the AIBA to enforce this rule universally by 2018.

## Methods

Data was collected through a voluntary, open-access, online poll. Detailed information and links to the poll were emailed to the National and Provincial Sport Organizations (PSO) for amateur boxing within Canada. The organizations were requested to disseminate the poll and information to their members. When club and coach emails were publicly listed on the respective PSO websites, they were sent an email describing the study and asked to disseminate the poll to their members. Participation was completely anonymous. The poll was available in English and French due to the bilingual character of Canada. A total of 257 organizations, club and coaches were contacted. Responses were collected over a period of 50 days (February 1, 2015 to March 23, 2015).

### Poll Details

The poll consisted of one required question and four optional questions. The required question—“In amateur boxing headgear should…”—was listed with five possible responses displayed in random order: (1) “…always be worn in all competition”; (2) “…never be worn by any competitor”, along with three conditional responses; (3) “…not be worn by men with more than 10 fights”; (4) “…not be worn by men in international competition”; and (5) “…Other” (for which the respondent could identify a specific preference). Response options 3 and 4 reflect the application of the headgear rules in Canada since September 2013 (no. 4) and the current modification since September 2014 (no. 3). The four optional questions provided demographic details comprising sex, age, region and role within the boxing community (i.e. active boxer, retired boxer, coach, parent of boxer, official, administrator, fan, or other—in which the respondent could provide their own response). The respondents were able to optionally submit comments and provide feedback. An overview of the comments commensurate to their frequency and content was selected to give a balanced representation of respondent feedback.

### Anonymity, Discarded Responses

To ensure anonymity, the IP address was not recorded. To track repeated responses, each submission was time stamped. Responses that had the identical comment and duplicate responses with overlapping characteristics within seconds or minutes of one another were flagged. In cases where a submission was identified as a repeat but with additional demographic information, only the most complete response was counted. Two scorers evaluated this independently. A total of 77 (12.1 % of the total) responses were determined to be repeat entries and discarded. The decision to discard or keep entries was made with high inter-rater reliability (Cohen’s kappa, *K* = 0.92).

### Estimating Community Opinion

To estimate a representative sample, a breakdown of membership to Boxing Canada by sex, region, class of active boxer by age and role (e.g. coach, official) was obtained from the Quebec Boxing PSO. From this data, we established a proportional breakdown of the boxing community’s demographic. In the poll, the demographic results provided a distribution for our sample. To calculate the representative opinion, we weighted the response distribution in the different demographics of our sample (e.g. senior female active boxers) by the proportion of the actual membership within the community. In the case of boxers 16 years of age or under, we weighted their membership proportion by the parents of boxers’ response distribution. For *junior* boxers (17–18 years), we weighted their membership proportion by the response distribution of active boxers under the age of 19. Based on this, a weighted estimate representative of the boxing community was possible.

### Multiple Membership Roles

Respondents to the survey were allowed to identify themselves with multiple roles in the boxing community (e.g. coach and active boxer). This reflects the targeted population, as individuals can hold multiple memberships in different categories. To mitigate the effect a lack of independence in relation to role might have on our analysis, we investigated the factors that were not impacted by multiple counts (age, sex and geographic region) separately from our analysis of role.

### Statistical Tests

Goodness-of-fit tests were performed using exact multinomial or binomial tests wherever possible. Tests for independence of dependent variables and response were performed using chi-square tests for independence. When cell counts were too small (<5) to use regular chi-square tests, we applied Monte Carlo simulation methods. In addition, *p* values were adjusted using the Holm adjustment method [15] to account for the fact that several tests for independence were performed in order to determine which variables significantly affected the response. Contingency tables were partitioned using the Agresti method [16]. Confidence interval estimates were calculated using the Goodman method [17, 18].

### Ethics

Institutional review board’s approval was not sought due to restrictions placed on doctoral students submitting protocols independently; however, the study was otherwise conducted in accordance with the Declaration of Helsinki [19]. Subjects were informed of the purpose of the survey before participating, and consent was inferred from the subjects’ voluntary, unpaid and anonymous participation.

## Results

**Fig. 1:**
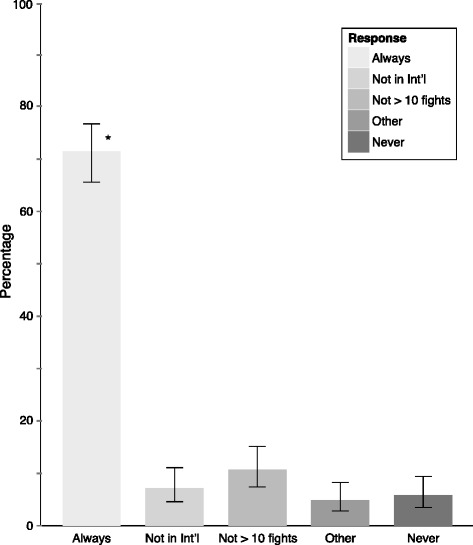
Canadian response distribution. Percentage distribution Canadian respondents provided to the question: “In amateur boxing headgear should be worn…”. The conditional options *Not in Int’l* and *Not >10 fights* refer to male boxers only to follow current rules. *p < 0.001, compared with all other options combined

A total of 636 responses were submitted, with 77 responses discarded as repeat submissions. There was no significant difference between these response distributions (*p* = 0.24). An additional 128 responses from outside Canada were not included in this analysis. The response distribution of international respondents did not differ significantly (*p* = 0.29) from the Canadian sample. A demographic of Canadian respondents is listed in Table 1. Of Canadian respondents, 71.5 % believed that headgear should be mandatory, greater (*p* < 0.001) than all other respondents combined (Fig. 1). In contrast 5.8 % believed that headgear should be prohibited. The remaining 22.7 % supported conditional removal (7.2 % in international competition, 10.7 % in greater than 10 fights and 4.9 % chose other). The complete voting distribution based on demographic is presented in Table 2.

**Table 1:** Respondent demographics

	Canada	West	Ontario	Quebec	East
Total	431	92	112	205	22
Sex					
Male	310	66	81	149	14
Female	116	24	29	55	8
Unlisted	5	2	2	1	0
Age					
≤18	32	4	4	22	2
19–40	236	42	60	124	10
>40	160	45	47	59	9
Unlisted	3	1	1	0	1
Role					
Active boxer	154	21	36	91	6
Retired boxer	113	34	37	32	10
Parent of boxer	53	18	16	14	5
Coach	169	44	58	54	13
Official	41	7	20	10	4
Administrator	20	10	7	1	2
Fan	44	13	15	13	3
Other	9	3	4	2	0

The Canada column is the sum of all regional columns

**Table 2:** Canadian response distribution

	Total (*n*)	Always % (standard error)	Not in international competition	Not >10 fights	Other	Never
Total	431	71.5 (5.6)	7.2 (3.2)	10.7 (3.8)	4.9 (2.7)	5.8 (3.0)
Sex						
Male	310	64.2 (6.9)	8.7 (4.2)	13.9 (5.1)	5.8 (3.5)	7.4 (3.9)
Female**	116	90.5 (7.2)	2.6 (4.5)	2.6 (4.5)	2.6 (4.5)	1.7 (4.0)
Unlisted	5	80 (34.7)	20 (34.7)	0 (28.5)	0 (28.5)	0 (28.5)
Age						
≤18	32	53.1 (20.7)	15.6 (16.2)	12.5 (15.1)	9.4 (13.9)	9.4 (13.9)
19–40	236	68.6 (7.7)	5.5 (4)	14 (5.8)	5.1 (3.8)	6.8 (4.3)
>40	160	78.8 (8.2)	8.1 (5.7)	5.6 (4.9)	3.8 (4.2)	3.8 (4.2)
Unlisted	3	100 (34.4)	0 (34.4)	0 (34.4)	0 (34.4)	0 (34.4)
Role						
Active boxer*	154	58.4 (10.0)	9.7 (6.3)	18.8 (8)	7.1 (5.5)	5.8 (5.1)
Retired boxer	113	77.9 (9.9)	6.2 (6.2)	7.1 (6.5)	4.4 (5.5)	4.4 (5.5)
Parent of boxer	53	86.8 (12.0)	5.7 (9.2)	3.8 (8.2)	1.9 (7.0)	1.9 (7.0)
Coach	169	74.6 (8.5)	5.9 (4.9)	7.7 (5.4)	5.3 (4.7)	6.5 (5.1)
Official	44	77.3 (15.6)	4.5 (9.6)	9.1 (11.7)	0 (6.6)	9.1 (11.7)
Administrator	41	82.9 (14.8)	7.3 (11.4)	4.9 (10.2)	4.9 (10.2)	0 (7.0)
Fan	20	75 (22.5)	5 (15.6)	10 (18.0)	5 (15.6)	5 (15.6)
Other	9	88.9 (26.3)	0 (21.2)	11.1 (26.3)	0 (21.2)	0 (21.2)

The confidence interval is asymmetric; hence, the standard error listed represents half the length of this interval. Demographics with few respondents result in large error estimates

*p < 0.01; **p < 0.001

### Determining Factors

**Fig. 2:**
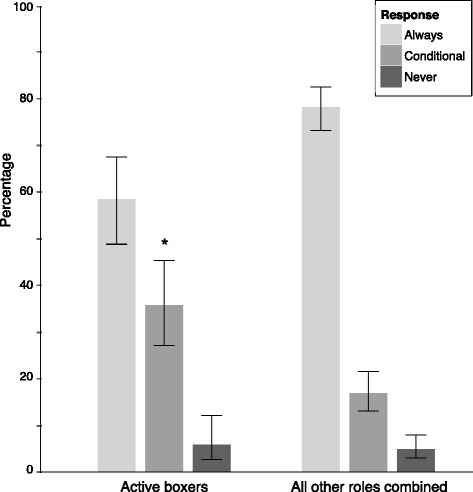
Response distribution of Canadian active boxers versus all other roles. Comparison of response distribution of Canadian active boxers to all other Canadian respondents combined, to the question: “In amateur boxing headgear should be worn…”. The *conditional* option refers to the sum of each conditional wearing of headgear response (i.e. “Not in international”, “Not >10 fights” and “Other”). *p < 0.001, when compared to the aggregate conditional response of all other respondents (using the Agresti method)

Within Canada, the sex and role of the respondent were determining factors for the response distribution. Females were significantly more in favour of mandatory headgear use and more against the banning of headgear (*p* < 0.001, corrected). Partitioning the contingency tables, we found that both male and female active boxers were significantly more likely to support the conditional wearing of headgear, as compared to respondents of the same sex in all other roles (*p* < 0.001 and *p* < 0.05, respectively). The proportion of active boxers favouring always versus never wearing headgear were not significantly different to those in other roles (*p* = 0.27) and, similarly, when tested for males and females separately (Fig. 2). When active boxers were excluded from the analysis, we found no significant difference in relation to role (*p* = 0.49). There was no significant difference across regions (*p* = 0.96, corrected) or age groups (*p* = 0.11, corrected). A breakdown by age and sex for active boxers is listed in Table 3.

**Table 3:** Canadian active boxer response distribution, by age and sex

	Total (*n*)	Always % (standard error)	Conditional	Never
Total	154	58.4 (9.3)	35.7 (9.1)	5.8 (4.7)
Sex				
Male	109	48.6 (11.2)	44 (11.1)	7.3 (6.2)
Female	44	81.8 (13.6)	15.9 (13.0)	2.3 (7.5)
Unlisted	1	100 (42.6)	0 (42.6)	0 (42.6)
Age				
≤18	27	51.9 (20.9)	40.7 (20.6)	7.4 (13.3)
19–40	115	57.4 (10.8)	36.5 (10.5)	6.1 (5.6)
>40	11	81.8 (25.1)	18.2 (25.1)	0 (17.1)
Unlisted	1	100 (42.6)	0 (42.6)	0 (42.6)

The confidence interval is asymmetric; hence, the standard error listed represents half the length of this interval. Demographics with few respondents result in large error estimates

### Other Respondents

Officials, parents of boxers and ringside physicians were almost unanimously against the banning of headgear, with 86 % believing in mandating headgear. Optional responses fell into three categories: (1) one quarter supported equal interpretation between the sexes, (2) half supported variations of removing headgear during international competition only and (3) one quarter supported headgear removal for adult boxers only.

### Representative Estimate

**Fig. 3:**
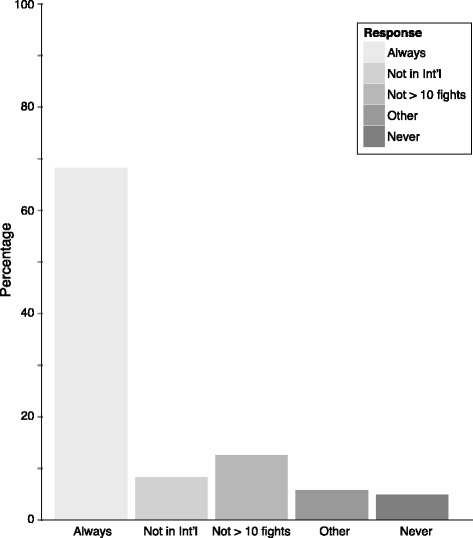
Estimated representative response distribution of Canadian boxing community. Representative opinion of the Canadian boxing community in response to the question: “In amateur boxing headgear should be worn…”. These values are estimated based on the response distribution for each respondent demographic, weighted by the corresponding proportional membership in amateur Canadian boxing. The opinion in favour of mandatory headgear is 68.3 %; of removing headgear only in international competition, it is 8.3 %; of removing headgear only for men with more than 10 fights, it is 12.6 %; of other conditional removal options, it is 5.8 %; and sentiment in favour of headgear prohibition is 4.95 %

The opinion representative of the Canadian boxing community was calculated as described in the “Methods” section (Table 4). This representative opinion was in accordance with the overall poll results with an estimated 68.3 % of the Canadian boxing community supporting mandatory headgear, less than 5 % for its prohibition and 12.6 % supporting the status quo (Fig. 3).

**Table 4:** Proportion of respondents versus (weighting by proportional membership)

	Parents	(Cadets)	Active boxer <19 years	(Junior)	Active boxer >19 years	(Senior)	Coach	(Coach)	Official	(Official)
Males	0.13	(0.23)	0.06	(0.11)	0.21	(0.28)	0.33	(0.16)	0.07	(0.05)
Females		(0.04)	0.01	(0.02)	0.1	(0.07)	0.08	(0.02)	0.02	(0.02)

The values are used to calculate the representative opinion of the Canadian boxing community. Membership data is based on 2006–2007 values. Parents are scored once, since the sex of the parent would not determine the outcome based on sex of the *cadet* boxer. Cadet boxers are <17 years old. Junior boxers are 17 or 18 years old. Headings not in brackets represent respondent demographic proportions, whereas headings in brackets represent actual membership proportions

### Feedback

Comments left by respondents described experiences of boxing with and without headgear from a personal perspective. The opinions of parents, coaches, officials and ringside physicians were also provided (Table 5). The comments selected provide an overview of the prevailing opinions, in proportion to their frequency. The majority of comments (80 %) were in support of headgear use, while 11 and 9 % of the comments were in support of the conditional use or the banning of headgear, respectively.

**Table 5:** Selected respondent comments

No.	Sex	Role	Comment
1	Male	Official, parent of boxer	“AIBA is not being genuine here. Their data is a load of nonsense. This change was and effort to make boxing more exciting for the fans. Nothing to do with safety. Ridiculous!!!”
2	Male	Retired boxer	“(At the) very least it should be a choice. But wearing headgear allows individuals to decide whether this sport is for them. Otherwise many will not endeavour to join clubs and try it out. This decision could be the death of the sport in Canada.”
3	Female	Active boxer	“Too many headbutts and injuries for amateur boxing. Every fight i go to there is at least one stoppage due to headbutt. Our athletes aren’t ready for it yet. Also, I don’t think fans would like to see females and kids getting hit without headgear so having them fight without it would not raise the numbers of participants in the sport. Please keep headgears for everyone!”
4	Male	Active boxer	“It should be up to the boxers and the coach whether they want to wear headgear. However headgear should be mandatory until fighters have developed defensive prowess.”
5	Female	Parent of boxer	“My son is a boxer with 15 fights. He’s only 18 but he’s been boxing since he was 10 years old. He’s already had 3 fights without headgear. Already received 2 headbutts, cuts and ecchymosis. He has great boxing talent, but due to this rule he’s retired from the sport.”^a^
6	Male	Retired boxer, coach	“Anything that restricts peripheral vision is dangerous.”
7	Male	Retired boxer	“All research indicates wearing headguard is safer. Also reduces cuts. Anyone complaining about vision isn’t wearing the correct size, or isn’t adjusting it correctly.”
8	Male	Coach	“Headgear for cuts only, so ‘vas’ up the face and gloves up, diving in for that hook to the body. IMO headgear increases concussive risk (larger target area, peripheral vision interference, merely transfers concussive force through 3/4 inches of dense foam).”
9	Male	Retired boxer, coach, official, administrator	“AIBA should not be allowed to dictate the use of head guards for competitions outside International competitions (multi) that they have jurisdiction over. Protection of boxers is paramountly important. Canada has been a leader in this field and were the main driving force behind bringing competitive head guards to the world of boxing. We all know that this is a campaign by AIBA to professionalize our amateur sport in an effort to control the sport of boxing, both amateur and professional world wise. Of course money is at the root of this move.”
10	Male	Coach	“Why risk injury to these young athletes? Should we not do everything we can to lessen the chance of injury? I think it is irresponsible to put our boxers in the ring without all the protection we can.”
11	Male	Coach	“When the boxers’ safety is traded for money.”
12	Female	Retired boxer, coach	“If this rule is implemented it will be next to impossible to get kids to register in our sport as it will scare parents away. I would not allow my own child to box without headgear. It will be the death of amateur boxing.”
13	Male	Coach, parent of boxer	“I believe that if the AIBA does away with headgear for all boxers at all ages it will kill the sport. I am a level 3 coach with 30 plus years of experience which includes over 16 years of competition, I also have two of my children involved in boxing and if the NO Headgear rule passed I would take my children out of the sport. I believe so would a lot of other parents.”
14	Male	Ringside physician	“With concussion information out and law suits in hockey and football, amateur boxing has been spared. BUT, box without headgear and law suits and injury will occur!!!! Second concussion syndrome in boxers under 19 years old means no physician in North America will be covered by insurance if anything happens and the second concussion syndrome occurs (death of the athlete). If you want to go underground, then do this. Lawyers will have a heyday with this!!! You will loose the few physicians you have.”

^a^Translated from French to English by PD

## Discussion

In this study, we measured the support within the Canadian boxing community for headgear use in amateur boxing through a voluntary, open-access online poll. To the best of our knowledge, this is the first study that has looked at the opinion of the boxing community in regard to headgear rules. Our results demonstrate that the majority (71.5 %) of Canadian respondents oppose any removal of headgear in competition while, in contrast, 5.8 % of respondents endorse prohibition of headgear. The estimated representative opinion of the Canadian boxing community was distributed similarly.

### Women in Amateur Boxing

Over 50 times as many women polled in favour of mandatory headgear use in all competition, as opposed to its prohibition. Possible reasons for differences found between male and female responses include the fact female bouts have only been sanctioned by the AIBA since 1993, well after headgear was introduced at the amateur level [20]. As such, women have no history of competing in amateur boxing without headgear. In contrast, men’s professional boxing without headgear is ubiquitous. Another factor is men and women show differences in risk assessment [21]. Women were found to rate the probability of negative consequences in extreme sports significantly more likely than men [22], which is believed to explain in part a lower life expectancy in men [23].

### Active Boxers and the Influence of Role in Boxing

Active boxers supported the conditional use of headgear more than other respondents, a result most pronounced for males. Actively competing boxers may be responding in relation to the constraints of their situation. Since this study did not examine the preferences before and after the rule changes, this cannot be confirmed. In social psychology, however, the concept of *normative social influence* describes the tendency to conform to group norms in order to be accepted by it [24]. This would be particularly salient for active boxers that would need to accept the status quo to continue competition. Further, as importance increases for the individual, *social impact theory* suggests a greater influence to conform to group norms [25]. Likewise, with contradictory viewpoints on safety, boxers that believe headgear impedes vision may accept some prohibition, despite other safety concerns.

### Safety and Perception

Banning headgear, according to the AIBA, will increase the athlete’s safety [1, 26]. Our results indicate a discrepancy between this premise and the opinion of the Canadian boxing community (Table 5, no. 1). As a result, a number of respondents, including those amenable to removing headgear, voiced concern that prohibiting headgear would destroy the sport (Table 5, no. 2).

Indeed, although the science surrounding head injury and headgear use in sport is complicated, the rate of severe concussion, as graded by the American Academy of Neurology [27], was reduced dramatically (7.5 to 0.7 %) subsequent to mandated headgear use, the introduction of computer scoring and other rule changes in amateur boxing [8]. Of the few prospective studies that have examined injury in amateur boxing, few concussive injuries were observed while participants wore headgear [28, 29]. This type of study has yet to be replicated without headgear. In contrast, the incidence of head injury observed in professional boxing, the model increasingly followed by amateur boxing, is high [30, 31].

Differing definitions of concussion, measurement methods [32] and variable symptom presentation [33] may also mean concussion rates are often underestimated. Since concussion does not require a knockout to occur [34], there may be a vast subset of undocumented brain injury, such as when concussive injury is not detected during the match [35, 36], traumatic brain injury [37] and chronic traumatic encephalopathy [38]. A systematic review of chronic traumatic brain injury risk in amateur boxing found most studies to be poor in quality [39]. Nonetheless, studies reviewed since 1990, after headgear use was made mandatory, listed far fewer abnormal observations than in earlier studies [39]. The need for prospective studies that examine boxers and outcomes longitudinally both in and out of the ring is integral to reasonably address safety.

The need to protect younger, inexperienced athletes through additional safety measures was raised by many respondents (Table 5, no. 3). This concern was pervasive even with respondents supportive of headgear removal in more experienced athletes (Table 5, no. 4). The vast majority of fights are contested at this novice level, yet the studies presented by the AIBA and the preponderance of boxing-based research conversely focus on outcomes in elite male athletes. Studies from other sports, however, show the effects of concussion on children to be much more severe than for adults [40]. This increases the risk for numerous developmental and later life problems, in particular following multiple concussions [41]. Similarly, women are more prone to concussion than men [41, 42]. Women suffer more severe symptoms, subjectively experiencing the post-concussion effects more acutely [43].

Safety concerns pertained as much to facial cuts as other more serious head injuries (Table 5, no. 5). Significantly more cuts occur without headgear [8, 44]. In the first tournaments without headgear, this was highlighted by controversy that followed athletes being disqualified with facial lacerations [45]. Subsequently, the AIBA has mandated the use of *Cavilon* (see rule 20.1.1) [2] as a preventative measure [46]. Further, without headgear, there can be a greater risk of brain injury from head clashes. This danger is outlined in a case report of acute subdural haematoma following competition [36]. This risk combined with the move to a professional model of scoring may lead to changes in ring strategy [36]. It is unclear how this may impact injury rates as boxers become accustomed to competing without headgear. In contrast, it has already been documented that the use of computer scoring led to a significant reduction in head injury [8].

For those supporting headgear removal, the predominant concern despite cuts was the danger of poor peripheral vision when wearing headgear (Table 5, no. 6); however, this was not unanimous (Table 5, no. 7). Although this concern has been noted previously in the literature [14], there has been no research into this possibility. There was also the belief that headgear did not offer a protective benefit, rather that the larger target increased concussive risk (Table 5, no. 8). Although studies do show a reduction of impact through headgear use [10], no studies that we are aware of have examined the impact a larger target area may have on injury risk.

### The Future of Amateur Boxing

Respondents to the poll often believed in a hidden agenda for the rule changes (Table 5, no. 9). This view has been reinforced through postings on a Canadian PSO website [47] as well as comments from current and former elite amateur boxers in the media [48]. In an editorial, McCrory et al. suggest that the professionalization of boxing may over the long term allow the AIBA to regulate boxing at all levels, from amateur to professional [14]. Many respondents perceived that the move to a more professional model was driven by money and expressed this as an abdication of the AIBA’s responsibilities (Table 5, nos. 10 and 11). Unlike professional boxing, however, there is no financial benefit to the amateur boxer to offset the risk of injury [49].

Doubting both the safety claims and the motivation behind banning headgear, many respondents questioned the impact this might have on future participation. These concerns centred on the refusal to allow children to continue with the sport without headgear (Table 5, no. 12). Respondents with a long history in boxing made similar claims (Table 5, no. 13). Without children entering the sport, long-term athlete development is threatened. Correspondingly, the greatest disapproval emanates from the building blocks of the sport: parents of boxers, officials and ringside physicians.

### Legal Implications

Collectively, questionable scientific evidence for and strong sentiment against banning headgear lead to a risk of lawsuit following severe injury, a concern listed by several respondents (Table 5, no. 14). Already in the USA, there are numerous lawsuits in other sports both at the professional and amateur levels that target how sport organizations manage head injury. More recent class-action lawsuits have targeted amateur sports including soccer [50], the National Collegiate Athletic Association [51] and state high-school organizations [52]. The underlying message is that sporting organizations are putting athletes at risk by not incorporating strenuous safety practices [50]. Seemingly in response to this concern, the USA appears to be the only country not currently following the AIBA’s headgear prohibition. Currently, American rules preclude boxing without headgear for safety reasons [53]. This inconsistency adds confusion to claims of greater safety from the AIBA.

### Limitations

The main limitation of this study was the sampling method. With a voluntary open-access, online poll, there is a potential bias based on accessibility. Not all boxing clubs or members have email resources. The organizations, clubs and coaches contacted may disseminate the poll to their members to varying degrees or not at all. This obstacle prevents us from accurately assessing the response rate. Only those motivated to respond would do so; however, due to the polarizing nature of the debate, we postulate equal motivation to respond independent of opinion. Conversely, the design addresses concerns of anonymity. Thus, the current methodology gave us access to a difficult-to-identify community, controlled for a response bias and consequently allowed us to estimate opinion within the community. Overall, the sample was large enough to calculate opinion within the Canadian boxing community; however, some of the demographics were underrepresented in the sample. Despite this, there were no significant regional response differences. We do not believe this had any significant impact on the results.

## Conclusions

In a Canadian sample of the amateur boxing community, the consensus supports mandatory headgear use. There is very little support for the banning of headgear proposed by the AIBA for 2018. Based on comments left by respondents, questions of safety and the perception of risk underline this sentiment. Respondents did not believe that removing headgear would make the sport safer. Corroborating this perception, the studies that do exist on headgear use in amateur boxing demonstrate a protective benefit. Active boxers, although more supportive of the conditional use of headgear, remained significantly against its prohibition. Parents, doctors and officials were almost unanimously against banning headgear. Continued scientific study on safety related to headgear use in boxing is essential. Prospective studies that observe boxers longitudinally, in and out of competition, would be of significant value. With concussion-related injury at the forefront of discussion and the opinion of the Canadian amateur boxing community strongly in favour of using headgear, there is currently little justification or evidence-based research to support the removal of headgear in amateur boxing.
